# Therapy satisfaction and willingness-to-pay in Polish patients with restless legs syndrome

**DOI:** 10.1007/s11325-021-02440-x

**Published:** 2021-08-01

**Authors:** Mariusz Sieminski, Marcelina Skrzypek-Czerko, Łukasz Chełminiak

**Affiliations:** 1 Department of Emergency Medicine, Medical University of Gdansk, Smoluchowskiego 17 , 80-214 Gdansk, Poland; 2 Department of Neurological-Psychiatric Nursing, Medical University of Gdansk, Skłodowskiej-Curie 3a, 80-210 Gdansk, Poland; 3 Department of Cardiology, Regional Hospital Grudziadz, Rydygiera 15/17, 86-300 Grudziadz, Poland

**Keywords:** Restless legs syndrome, Quality of life, Patient satisfaction, Willingness to pay

## Abstract

**Purpose:**

Restless legs syndrome (RLS) is a serious burden for patients which can be measured in economic terms by assessing the money spent on therapy and the willingness to pay. The aim of this study was to assess whether or not patients feel satisfied with the therapy relative to the money they spent on the treatment, and to assess patients’ willingness to pay for therapy that fully eliminates all RLS symptoms.

**Methods:**

Adult subjects with RLS confirmed by actual international consensus criteria, a positive RLS-Diagnostic Index (RLS-DI) score, and clinical examinations and observations were assessed to generate a disease severity index. An original set of questions was used to collect data on patient satisfaction with therapy and their willingness to pay.

**Results:**

Among 100 subjects, 27% were not satisfied with therapy; this subgroup was characterized by lower indices of severity of the disease. Patients spent approximately 3% of their income in treating RLS. They are willing to pay up to 8.3% of their income to eliminate symptoms.

**Conclusions:**

The cost of RLS therapy is a significant part of patient expenditure. Nevertheless, RLS may at times remain so troublesome for patients that they are willing to spend more on therapy to eliminate symptoms.

## Introduction

The prevalence of restless legs syndrome (RLS) is 7–10% of the general population in Western countries [1] However, RLS remains largely neglected despite its significant negative effect on patients’ quality of life [2]. RLS is a sensorimotor syndrome presenting with an urge to move the legs, accompanied by an unpleasant sensation in the lower extremities. The symptoms appear during periods of rest (e.g., while sitting or lying), especially in the evening and at the night. They typically disappear when the patient starts to move their legs and reappear immediately after stopping the movements [3]. Due to the diurnal rhythm of symptoms, RLS frequently causes sleep disorders, mostly insomnia with delayed sleep onset or problems with sleep maintenance. Symptomatic therapies of RLS are available. The reduction of symptoms has been proven in numerous clinical trials with dopaminergic antagonists [4], alfa2delta ligands [5], and opioids [6].

The efficacy of treatment is usually evaluated using scales that assess the severity of symptoms and quality of life. These variables are crucial for assessing drug efficiency; nevertheless, such data collected during a clinical trial may differ from real-life experiences. There are two important issues related to the treatment of RLS patients that must be addressed. The first is general patient satisfaction with the treatment. This is an important factor influencing patient adherence [7] as complications can arise during therapy. The most important and specific complication is augmentation, which increases the severity of symptoms related to the appropriate therapy [8]. Another issue is the relationship between patient costs related to pharmacotherapy and patient satisfaction with treatment results relative to money spent on the drug. The economic cost of RLS therapy is a significant burden on patients [9] and is comparable with other chronic conditions, such as diabetes [10, 11].

The cost-effectiveness of RLS therapy has been proven in terms of quality-adjusted life years for dopaminergic therapy [12]. However, patients may not consider RLS treatment as necessary. RLS is frequently neglected as a disorder as it does not shorten life expectancy nor does RLS commonly lead to severe disability. Therefore, while it is difficult to judge the importance of a disease to a patient, some conclusions may be drawn from patients’ declarations of how much money they are ready to spend on treatments—their so-called willingness-to-pay (WTP). However, data on RLS patients’ WTP and issues related to their treatment are lacking. This study therefore aims to (1) establish whether patients were satisfied with RLS treatment in the context of its costs; and (2) assess the WTP of patients with RLS.

## Materials and methods

### Patients

Adult patients with RLS diagnosed according to consensus criteria [3] were recruited for this study. The sample consisted of patients with positive results from the RLS-Diagnostic Index (RLS-DI) questionnaire [13] (RLS-DI score greater than 11) and with proven efficacy of dopaminergic therapy. They had to have remained under clinical observation for at least 6 months (with at least two outpatient visits) with available and complete clinical data about the severity of their RLS during the diagnosis and follow-up. Patients with cognitive disorders that interfered with communication and those who did not consent to the collection of data for this study were excluded. All patients were from the same tertiary center and were diagnosed, treated, and regularly assessed by the same RLS expert. The patients were paying for the therapy by themselves because drugs used in RLS therapy are not reimbursed in Poland.

### Study design

Patient demographics (age, sex, and comorbidities) and clinical data regarding patients’ status before the initiation of therapy were retrospectively obtained from their medical records. During an outpatient visit devoted to this study, an actual clinical study was evaluated using clinical scales that assessed the quality of life. Patients were asked whether they agreed to participate in a telephone interview about their satisfaction with the treatment and WTP. Those who agreed were contacted by a member of the study team in charge of collecting the information. The data were gathered between March and June 2017.

As the process of acquiring data from the subjects was not anonymized, we refrained from direct questions about an individual’s monthly income. To estimate the income in the studied group, we decided to assign the average salary to each fully employed participant and the average pension to each retired patient. On June 30, 2017, the mean salary in Poland was 4,508.08 PLN (1,066.62 Euros) (www.stat.gov.pl), and the exchange rate of the Polish zloty (PLN) to 1 Euro was 4.2265 PLN (www.nbp.pl). The mean pension during this period was 2,085.60 PLN (www.zus.gov.pl). The study protocol was approved by the Bioethical Committee of the Medical University of Gdansk.

### Assessment of clinical status and therapy satisfaction

The clinical condition of patients was assessed during the diagnostic visit (i.e., before the initiation of therapy) and during the study visit. The severity of RLS was assessed using the International RLS Severity Scale (IRLS) [14]. Daytime sleepiness was assessed using the Epworth Sleepiness Scale (ESS) [15] and the severity of insomnia was assessed using the Athens Insomnia Scale (AIS) [16].

Quality of life was assessed using a validated version of the EQ-5D scale [17], which consists of two components. The first is a visual analog scale ranging from 0 (death) to 100 (best imaginable health condition) on which patients were asked to mark their condition before therapy and at the time of examination. The second component is more descriptive; patients were asked to assess their situation in five domains: mobility, self-care, daily activities, pain/discomfort, and anxiety/depressed mood. For each domain, patients may grade themselves with 1 to 5 points, with 1 point signifying a lack of any problems within a domain and 5 points signifying severe problems in one domain. The raw results of this part of the questionnaire are presented as a combination of five digits (starting from “11,111” meaning no problems in any of the domains and ending with “55,555” meaning extreme problems in all domains). This allowed us to define 3,125 possible health states, each of which may be converted into a single value using a set of values derived for the general population of a specific country. The set of values, ranging from − 0.523 for worst health condition (health state of “55,555”) to 1.0 for best health condition (health state of “11,111”), was developed for the Polish population [17].

Satisfaction with and the financial burden of the treatment, as well as WTP were assessed using the following set of questions:

1. Are you satisfied with the improvement in sleep quality after therapy? (YES/NO)
2. Are you satisfied with symptom control after therapy? (YES/NO)
3. Are you satisfied with the improvement in your everyday functioning after therapy? (YES/NO)
4. How much money do you spend monthly on the treatment of RLS?
5. Are you satisfied with the results of the therapy in terms of the money you spent? (YES/NO)
6. How much money would you be willing to spend monthly to control your symptoms through RLS therapy?
7. How much money would you be willing to pay for a single procedure (e.g., surgical) leading to a complete control of the symptoms?

Statistical analysis was performed using the *t*-test for continuous variables, and chi-square tests for discrete data. Correlations were assessed using Pearson’s correlation coefficient calculations. Statistical significance was set at *P* < 0.05.

## Results

A total of 100 RLS patients (36 men) were recruited for this study. The mean age of the group was 66.4 years, with an average of 15 years living with restless legs syndrome. The mean duration of clinical observation was 35.3 months. Of the subjects, 41% had at least one comorbidity, with arterial hypertension (35%), cardiovascular diseases (10%), and depression (17%) being the most frequent. Most patients were treated with monotherapy (ropinirole, 64%; l-Dopa, 5%; pregabalin, 6%), while 23% were treated with combined therapy (ropinirole + pregabalin; ropinirole + l-Dopa). Over one-third of the subjects were fully employed and 62% were retired with an approximate mean monthly income of 3,014.22 PLN (713.17 Euros).

The therapy conducted resulted in a significant improvement in all clinical and quality of life scales, as shown in Table 1.

**Table 1:** Results of the therapy

Scale	Result before therapy (Mean, SD)	Result at the moment of the study (Mean, SD)	*p*
IRLS^a^	21.5 (4.7)	9.1 (4.0)	< 0.005
AIS^b^	12.6 (2.5)	8.6 (1.0)	< 0.005
ESS^c^	9.8 (1.9)	6.7 (1.7)	< 0.005
EQ-5D VAS (0–100)^d^	47.1 (22.6)	68.2 (19.8)	< 0.005
EQ-5D descriptive^e^	0.70 (0.2)	0.89 (0.1)	< 0.005

^a^International RLS Severity Scale

^b^Athens Insomnia Scale

^c^Epworth Sleepiness Scale

^d^Visual analog scale of EQ-5D scale

^e^Descriptive part of EQ-5D scale

**Fig. 1:**
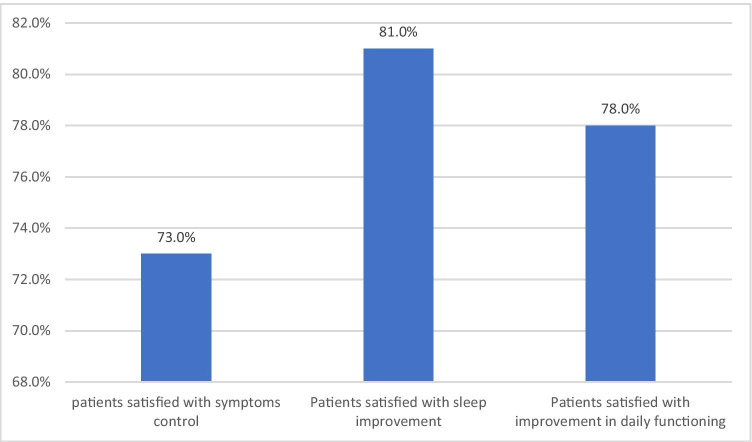
Percentage of patients satisfied with therapy

Among the subjects, 73% were satisfied with the treatment results in terms of symptom control, 81% reported satisfaction with sleep improvement, and 78% were satisfied with the improvement in their functioning in everyday life (Fig. 1).

The percentage of satisfied and unsatisfied patients differed significantly in terms of the initial and final IRLS score, final ESS score, EQ-5D VAS score, and EQ-5D descriptive component score. Satisfied patients were characterized by significantly higher initial IRLS scores (24.9% vs. 20.2%, *p* < 0.005), higher final IRLS (11.0% vs. 8.4%; *p* = 0.024), and ESS scores (7.6% vs. 6.3%, *p* = 0.004). They also had lower final scores on the EQ-5D scale. The results are presented in Table 2.

**Table 2:** Results on clinical scales in groups of patients satisfied and not satisfied with treatment results

Scale	Patients satisfied with therapy (*n* = 73) (Mean, SD)	Patients not satisfied with therapy (*n* = 27) (Mean, SD)	*p*
IRLS^a^ before therapy	24.9 ± 3.3	20.2 ± 4.6	< 0.005
IRLS at the moment of study	11.0 ± 3.8	8.4 ± 3.9	0.024
AIS^b^ before therapy	13.25 ± 2.2	12.4 ± 2.6	0.263
AIS at the moment of study	9.0 ± 1.2	8.45 ± 0.9	0.073
ESS^c^ before therapy	10.4 ± 1.5	9.6 ± 2.1	0.159
ESS at the moment of study	7.75 ± 1.4	6.3 ± 1.7	0.004
EQ-5D VAS^d^ (0–100) before therapy	48.4 ± 24.5	48.2 ± 21.3	0.48
EQ-5D VAS (0–100) at the moment of study	51.6 ± 22.0	74.3 ± 15.1	< 0.005
EQ-5D descriptive^e^ before therapy	0.67 ± 0,21	0.71 ± 0.19	0.21
EQ-5D descriptive at the moment of study	0.82 ± 0.16	0.92 ± 0.06	< 0.005

^a^International RLS Severity Scale

^b^Athens Insomnia Scale

^c^Epworth Sleepiness Scale

^d^Visual analog scale of EQ-5D scale

^e^Descriptive part of EQ-5D scale

The mean monthly cost of therapy was 90.00 PLN (21.3 Euros). This amount constitutes 3.0% of the approximate average monthly income in the examined group.

Eighty-two patients reported satisfaction with the results of therapy in terms of the costs. Patients who were unsatisfied paid slightly more for their therapy (102.7 PLN vs. 86.7 PLN), although the difference was not statistically significant. Patients not satisfied with the relationship between therapy costs and its effects were characterized by a higher initial IRLS score (24.1% ± 4.2% vs. 20.1% ± 4.7%, *p* = 0.02), higher IRLS score during the duration of the study (11.3% ± 4.4% vs. 8.6% ± 3.8, *p* = 0.02), and lower EQ-5D descriptive component score (0.8 ± 0.2 vs. 0.9 ± 0.07, *p* = 0.01) after therapy. It is noticeable that subjects who were satisfied with the money spent on therapy initially had a lower quality of life (EQ-5D VAS 45.0 ± 20.1, vs. 62.7 ± 24.9, *p* = 0.007). Moreover, therapy resulted in significantly lower AIS scores in the not-satisfied group (8.7 ± 1.1 vs. 8.0 ± 0.1, *p* = 0.016). The differences between the two groups are presented in Table 3.

**Table 3:** Results on clinical scales in groups of patients satisfied and not satisfied with treatment results in terms of therapy costs

Scale	Patients satisfied with therapy (*n* = 82) (Mean, SD)	Patients not satisfied with therapy (*n* = 18) (Mean, SD)	*p*
IRLS^a^ before therapy	20.1 ± 4.7	24.1 ± 4.2	0.02
IRLS	8.6 ± 3.8	11.3 ± 4.4	0.02
AIS^b^ before therapy	12.6 ± 4.2	12.5 ± 2.5	0.450
AIS at the moment of study	8.7 ± 1.1	8.0 ± 0.1	0.016
ESS^c^ before therapy	9.7 ± 1.9	9.9 ± 1.8	0.409
ESS at the moment of the study	6.8 ± 1.8	6.1 ± 1.3	0.093
EQ-5D VAS^d^ (0–100) before therapy	45.0 ± 20.1	62.7 ± 24.9	0.007
EQ-5D VAS (0–100) at the moment of study	69.7 ± 18.6	61.8 ± 24.4	0.118
EQ-5D descriptive^e^ before therapy	0.68 ± 0.2	0.79 ± 0.18	0.062
EQ-5D descriptive at the moment of study	0.91 ± 0.07	0.83 ± 0.2	0.01

^a^International RLS Severity Scale

^b^Athens Insomnia Scale

^c^Epworth Sleepiness Scale

^d^Visual analog scale of EQ-5D scale

^e^Descriptive part of EQ-5D scale

**Fig. 2:**
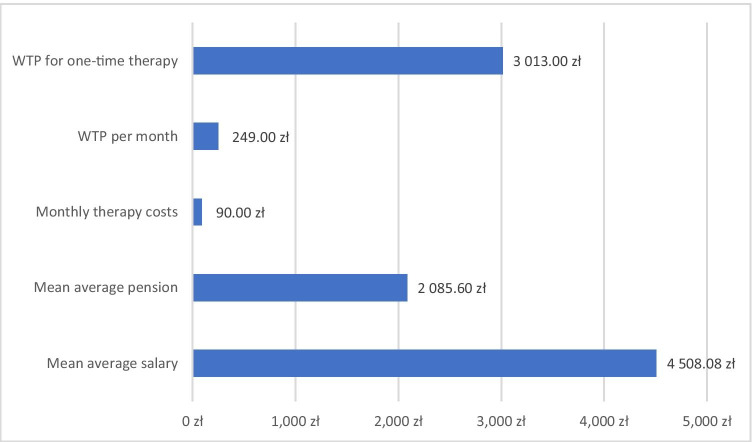
Cost of real therapy per month, monthly WTP for therapy to reduce the symptoms, and WTP for a one-time therapy to eliminate symptoms vs the average monthly salary and pension in Poland at the moment of performing the study

Patients declared that they were willing to pay 249 PLN (± 264.5 PLN) monthly to reduce the symptoms. This amount constitutes 8.26% of the approximate income within the group. The patient was willing to pay 3,013 PLN (± 3,187.4 PLN) for a single procedure to eliminate all symptoms. These values are compared with the average income of Polish patients in Fig. 2. We found that the WTP for monthly chronic therapy was significantly negatively correlated with the EQ-5D descriptive component initial score (before therapy), that is, the worse the quality of life, the more patients were ready to pay more for treatment (Pearson’s coefficient *r* =  − 0.3). Other clinical features of RLS did not correlate with WTP.

## Discussion

Patient satisfaction with therapy is a neglected issue. Clinicians participating in clinical trials focus on objective measurement (e.g., biochemical, radiological) and clinical scales (e.g., IRLSS), which are essential because proving that a substance does influence the course of the disease would be impossible without these measurements. Nevertheless, it must be remembered that patient satisfaction is not reflected in laboratory tests or even clinical scales. An assessment of the quality of life using increasingly sophisticated tools bridges the gap between medical results and patient satisfaction in many trials. It has also been performed in RLS trials [18–20]. Quality of life questionnaires bring useful data about interference between the disease, therapy, and the patient’s life, although they do not give information that could be obtained from directly asking patients (e.g., “Are you satisfied with the results of the therapy you have received?,” “Do you think your life has improved after relieving the symptoms with the pills we have given you?”). Our study notes that the unsatisfied population had better results in clinical scales than the satisfied patients. The gap between the improvement in clinical scales and satisfaction may suggest that there is a place for non-pharmacological strategies of therapy in RLS, by improving coping strategies, for example, as was suggested in a study by Hornyak et al. [21]. The percentage of patients declaring their satisfaction with RLS therapy was found to be higher than that of populations suffering from other chronic disorders, such as cluster headaches [22].

We also asked the patients whether they were satisfied with the improvement in their symptoms relative to the money they spent on therapy (“Was it worth it?”). To the best of our knowledge, such data in the field of RLS are unavailable, although recent studies on its socioeconomic consequences for European subjects showed that delayed diagnosis and erroneous therapy (factors certainly causing patient dissatisfaction) lead to increased socioeconomic costs of the disease [23]. We found that the patients who were not satisfied with the relationship between money spent and therapeutic effect had worse initial scores on RLS severity scales, and on the EQ-5D scale. These results are opposite to what was observed with the satisfaction regardless of costs. This may suggest that assessing patient satisfaction in the context of expenditures may provide a more precise assessment. Such data are scarce (both in RLS and other disorders) and results are contradictory; for example, Goldstein et al. found no relationship between the costs of care and patients’ satisfaction with tinnitus [24].

The data we collected allowed us to estimate the economic burden of RLS therapy for Polish households. The costs of therapy were equal to nearly 3% of the estimated income in the examined group, but the monthly cost should be analyzed in the context of disposable income (i.e., the amount of money left after paying taxes and social security charges). The average disposable income in Polish households in 2017 was 1,598 PLN *per capita* (www.stat.gov.pl) (379.1 Euros), and the average monthly expenditure was 1,176 PLN (278.24 euros) *per capita*. This means that the monthly cost of drugs for RLS constituted 5.6% of the average Polish disposable income and 7.6% of the average Polish monthly income. According to published data, health-related expenditures constitute approximately 5.5% of the expenditures of Polish citizens [25]. This means that therapy for RLS is a serious economic burden for the examined population—costs of its treatment consummated the amount of money routinely dedicated to all health expenditures in Polish households. The fact that 40% of the study’s participants had at least one comorbidity was especially striking—the comorbidities were naturally causing additional costs.

Analyzing the WTP in the context of national average disposable income *per capita* and monthly expenditures *per capita* allows us to more realistically present the financial burden caused by the disorder; subjects with RLS are ready to spend 249 PLN monthly for chronic effective therapy, which constitutes 15.6% of Polish disposable income per capita and 21.2% of monthly expenditures. The average declared payment for a one-time hypothetical cure bringing life-long freedom from symptoms was slightly over 3,000 PLN. This means that patients are prepared to sacrifice savings collected from 7 to 8 months (the difference between disposable income and expenditures) for such therapy. These data suggest that RLS is problematic for patients.

Data on WTP for sleep disorders are scarce, and publications on the topic have not been found in the field of RLS. Roy et al. found that patients with insomnia were ready to pay almost 67 US dollars to shorten their sleep onset latency by 10 min, reduce wake time after sleep onset by 15 min, or prolong their total sleep time by 1 h. These improvements are not significant, which shows the importance (with importance measured financially) of sleep disorders [26].

A recent study by Huang et al. showed that Australian citizens were ready to regularly pay about 3.9% of their household income to avoid any chronic disorders [27]. Patients in our study were ready to spend much more (15.6% of the average monthly disposable income) to be free from symptoms of RLS. This may be because the patient’s experience of RLS symptoms is really troublesome. Moreover, we found that WTP is related to quality of life. According to our data, the lower the quality of life, the higher the patient’s WTP for efficient therapy. Such a relationship has not been described in other chronic conditions, such as headaches [28]. One positive piece of information is that the costs of therapy reported by patients in our study were lower than their WTP, which is opposite to that observed in narcolepsy [29, 30].

Our study had some limitations. It must be remembered that participants were faced with highly theoretical situation of assessing their own willingness-to-pay. The solutions proposed to the participants of the study (regularly taken and completely efficient chronic therapy or single procedure removing the symptoms) do not exist. It is probably then that participants had overestimated their readiness to spend money—in real life, the amounts spent on such curations would be probably lower. The most important limitation is that we did not collect data on the individual income of the participants. We had to use general Polish statistical data to analyze the costs of therapy and WTP. This may have caused some bias in drawing conclusions about the study group. Nevertheless, comparing the economic data of our patients with national economic indicators allowed us to see RLS as an economic burden from a global perspective, as treatment costs are comparable across the country. Another limitation is the number of participants. Moreover, the percentage of patients unsatisfied with therapy (27%) left even smaller group of subjects to perform analysis of willingness-to-pay. Increasing the study group would allow us to draw stronger conclusions. The strength of our study lies in the selection of patients. All patients were examined and diagnosed with RLS on the basis of history, clinical diagnostic questionnaires (RLS-DI), and neurologic examination. They had a long history of clinical observation, so RLS mimics were excluded.

## Conclusions

The available treatment modalities allow a significant reduction in RLS symptoms, which leads to a noticeable improvement in patients’ quality of life. The cost of RLS therapy is an important part of patient expenditures. Nevertheless, RLS is so troublesome that patients are still willing to spend even more money on therapies to get rid of the symptoms. Future studies should compare different therapies in terms of patient satisfaction.

## Data Availability

Data are available from the authors upon request.

## References

[1] Allen RP, Walters AS, Montplaisir J (2005). Restless legs syndrome prevalence and impact: REST general population study. Arch Intern Med.

[2] Wesström J, Nilsson S, Sundström-Poromaa I, Ulfberg J (2010). Health-related quality of life and restless legs syndrome among women in Sweden. Psychiatry Clin Neurosci.

[3] Allen RP, Picchietti DL, Garcia-Borreguero D (2014). Restless legs syndrome/Willis-Ekbom disease diagnostic criteria: updated International Restless Legs Syndrome Study Group (IRLSSG) consensus criteria–history, rationale, description, and significance. Sleep Med.

[4] Scholz H, Trenkwalder C, Kohnen R, Riemann D, Kriston L, Hornyak M (2011) Dopamine agonists for restless legs syndrome. Cochrane Database Syst Rev (3):CD006009. 10.1002/14651858.CD006009.pub2 10.1002/14651858.CD006009.pub2 PMC8908466 21412893

[5] Allen R, Chen C, Soaita A (2010). A randomized, double-blind, 6-week, dose-ranging study of pregabalin in patients with restless legs syndrome. Sleep Med.

[6] Trenkwalder C, Beneš H, Grote L (2013). Prolonged release oxycodone-naloxone for treatment of severe restless legs syndrome after failure of previous treatment: a double-blind, randomised, placebo-controlled trial with an open-label extension. Lancet Neurol.

[7] Bakar ZA, Fahrni ML, Khan TM (2016). Patient satisfaction and medication adherence assessment amongst patients at the diabetes medication therapy adherence clinic. Diabetes Metab Syndr.

[8] García-Borreguero D, Williams A-M (2010). Dopaminergic augmentation of restless legs syndrome. Sleep Med Rev.

[9] Dodel R, Happe S, Peglau I (2010). Health economic burden of patients with restless legs syndrome in a German ambulatory setting. Pharmacoeconomics.

[10] Reese JP, Stiasny-Kolster K, Oertel WH, Dodel RC (2007). Health-related quality of life and economic burden in patients with restless legs syndrome. Expert Rev Pharmacoecon Outcomes Res.

[11] Reinhold T, Müller-Riemenschneider F, Willich SN, Brüggenjürgen B (2009). Economic and human costs of restless legs syndrome. Pharmacoeconomics.

[12] Lees M, Roberts G, Tabberer M (2008). Cost-effectiveness of licensed treatment options for restless legs syndrome in the UK and Sweden. Curr Med Res Opin.

[13] Benes H, Kohnen R (2009). Validation of an algorithm for the diagnosis of restless legs syndrome: the Restless Legs Syndrome-Diagnostic Index (RLS-DI). Sleep Med.

[14] Walters AS, LeBrocq C, Dhar A (2003). Validation of the International Restless Legs Syndrome Study Group rating scale for restless legs syndrome. Sleep Med.

[15] Johns MW (1991). A new method for measuring daytime sleepiness: the Epworth sleepiness scale. Sleep.

[16] Soldatos CR, Dikeos DG, Paparrigopoulos TJ (2000). Athens Insomnia Scale: validation of an instrument based on ICD-10 criteria. J Psychosom Res.

[17] Golicki D, Niewada M, van Hout B (2014). Interim EQ-5D-5L value set for Poland: first crosswalk value set in Central and Eastern Europe. Value Health Reg Issues.

[18] Winkelman JW, Sethi KD, Kushida CA (2006). Efficacy and safety of pramipexole in restless legs syndrome. Neurology.

[19] Högl B, Oertel WH, Stiasny-Kolster K (2010). Treatment of moderate to severe restless legs syndrome: 2-year safety and efficacy of rotigotine transdermal patch. BMC Neurol.

[20] Garcia-Borreguero D, Grunstein R, Sridhar G (2007). A 52-week open-label study of the long-term safety of ropinirole in patients with restless legs syndrome. Sleep Med.

[21] Hornyak M, Grossmann C, Kohnen R (2008). Cognitive behavioural group therapy to improve patients’ strategies for coping with restless legs syndrome: a proof-of-concept trial. J Neurol Neurosurg Psychiatry.

[22] Bekkelund SI, Ofte HK, Alstadhaug KB (2014). Patient satisfaction with conventional, complementary, and alternative treatment for cluster headache in a Norwegian cohort. Scand J Prim Health Care.

[23] Trenkwalder C, Tinelli M, Sakkas GK (2021). Socioeconomic impact of restless legs syndrome and inadequate restless legs syndrome management across European settings. Eur J Neurol.

[24] Goldstein E, Ho C-X, Hanna R (2015). Cost of care for subjective tinnitus in relation to patient satisfaction. Otolaryngol Head Neck Surg.

[25] Stanisławska J, Agnieszka K, Głowicka-Wołoszyn R (2018) Zmiany w poziomie i strukturze wydatków polskich gospodarstw domowych o różnej sytuacji dochodowej w aspekcie zrównoważonej konsumpcji. Handel Wewnętrzny 3(374):358–370

[26] Roy AN, Madhavan SS, Lloyd A (2015). A discrete choice experiment to elicit patient willingness to pay for attributes of treatment-induced symptom relief in comorbid. Insomnia Manag Care.

[27] Huang L, Frijters P, Dalziel K, Clarke P (2018). Life satisfaction, QALYs, and the monetary value of health. Soc Sci Med.

[28] Lampl C, Steiner TJ, Mueller T (2012). Will (or can) people pay for headache care in a poor country?. J Headache Pain.

[29] Bolin K, Berling P, Wasling P (2017). The cost-utility of sodium oxybate as narcolepsy treatment. Acta Neurol Scand.

[30] Bolin K, Niska P-Å, Pirhonen L (2020). The cost utility of pitolisant as narcolepsy treatment. Acta Neurol Scand.

